# Bosutinib in Japanese patients with newly diagnosed chronic-phase chronic myeloid leukemia: final 3-year follow-up results of a phase 2 study

**DOI:** 10.1007/s12185-022-03435-4

**Published:** 2022-08-13

**Authors:** Takaaki Ono, Masayuki Hino, Itaru Matsumura, Shin Fujisawa, Kenichi Ishizawa, Emiko Sakaida, Naohiro Sekiguchi, Chiho Ono, Mana Aizawa, Yusuke Tanetsugu, Yuichiro Koide, Naoto Takahashi

**Affiliations:** 1 Hamamatsu University Hospital, 1-20-1 Handayama, Higashi-ku, Hamamatsu, Shizuoka, 431-3192 Japan; 2 Osaka City University Hospital, Osaka, Japan; 3 Kindai University Hospital, Osaka, Japan; 4 Yokohama City University Medical Center, Yokohama, Japan; 5 Yamagata University Hospital, Yamagata, Japan; 6 Chiba University Hospital, Chiba, Japan; 7 National Hospital Organization Disaster Medical Center, Tokyo, Japan; 8 Pfizer Japan Inc, Tokyo, Japan; 9 Pfizer R and D Japan G.K, Tokyo, Japan; 10 Akita University Hospital, Akita, Japan

**Keywords:** Bosutinib, Tyrosine kinase inhibitor, Chronic myeloid leukemia, Japan

## Abstract

Bosutinib has been evaluated for treatment of chronic-phase chronic myeloid leukemia (CP-CML) in several clinical studies, including in Japan. This open-label, single-arm, phase 2 study evaluated the efficacy and safety of bosutinib at a starting dose of 400 mg once daily in Japanese patients (*n* = 60) with newly diagnosed CP-CML. The minimum follow-up period was 3 years and median duration of treatment was 35.9 months. At study completion, 60% of patients were still on treatment. Cumulative rates of major molecular response (MMR), molecular response^4^ (MR^4^), and MR^4.5^ at any time were 70.0%, 53.3%, and 48.3%, respectively. No patient who achieved MMR or MR^4^ had a confirmed loss of response. No patient experienced on-treatment transformation to accelerated/blast phase or died within 28 days of the last bosutinib dose. Any-grade treatment-emergent adverse events (TEAEs) occurred in 100% (grade ≥ 3: 81.7%) of patients. The most common TEAEs were diarrhea (86.7%), increased alanine aminotransferase (55.0%), and increased aspartate aminotransferase (46.7%). No new safety signals emerged during the follow-up period. Bosutinib continues to demonstrate a favorable benefit/risk profile and is an important treatment option for Japanese patients with newly diagnosed CP-CML. Optimal management of TEAEs during initial treatment with bosutinib should be prioritized.

Trial Registration: ClinicalTrials.gov ID: NCT03128411.

**Supplementary Information:**

The online version contains supplementary material available at 10.1007/s12185-022-03435-4.

## Introduction

Bosutinib is an oral *BCR*::*ABL1*-targeting tyrosine kinase inhibitor (TKI) approved in several countries, including Japan, as first-line therapy for adult patients with newly diagnosed chronic-phase chronic myeloid leukemia (CP-CML) at a starting dose of 400 mg once daily (QD) [1–3]. Bosutinib is also approved at a starting dose of 500 mg QD for the treatment of adult patients with Philadelphia chromosome–positive (Ph +) CP, accelerated phase (AP) or blast phase (BP) CML resistant or intolerant to prior therapy [1–3].

Bosutinib has been evaluated in several clinical studies [4–8], including in Japan [9, 10], for the treatment of CML. In the primary analysis of a phase 2 study of first-line bosutinib at a starting dose of 400 mg QD in Japanese patients with CP-CML, the major molecular response (MMR) rate at 12 months was 55.0%, which met the primary objective of the study [11]. Adverse events (AEs) were manageable and consistent with the known safety profile of bosutinib [11].

Here, we report the results of the final ≥ 3-year efficacy and safety follow-up of this phase 2 study, including data collected after the primary data cut off, which has been previously reported [11].

## Materials and methods

### Study design

This was an open-label, single-arm, phase 2 study (ClinicalTrials.gov ID: NCT03128411) to evaluate the efficacy and safety of bosutinib in Japanese adult patients with newly diagnosed CP-CML. The study was conducted in accordance with the Declaration of Helsinki, the International Council for Harmonisation Guidelines for Good Clinical Practice, and local regulatory requirements. The study protocol, protocol amendments, and informed consent documents (provided by all patients) were approved by the Institutional Review Board at each study center in Japan.

### Patients and treatment

Japanese patients were ≥ 20 years of age with a molecular diagnosis of CP-CML (detection of *BCR*::*ABL1* rearrangement) within the previous 6 months, an Eastern Cooperative Oncology Group performance status (ECOG PS) score of 0 or 1, and adequate renal and hepatic function; and had resolved acute effects of any prior therapy to baseline severity or grade ≤ 1. Patients were excluded if they had prior treatment for CML (except for hydroxyurea within 6 months); central nervous system involvement; extramedullary disease only; major surgery or radiotherapy within 14 days of registration; history of clinically significant or uncontrolled cardiac disease; active, uncontrolled bacterial, fungal, or viral infection; clinically significant gastrointestinal disorder; history of another malignancy within 5 years prior to registration (except for basal cell carcinoma or cervical carcinoma in situ or stage 1/2 cancer in complete remission for ≥ 12 months); or uncontrolled hypomagnesemia or uncorrected hypokalemia.

Patients received bosutinib at a starting dose of 400 mg QD. Dose escalation to a maximum of bosutinib 600 mg QD was permitted for patients with unsatisfactory response (e.g., *BCR*::*ABL1* transcripts > 10% and/or Ph + metaphases > 35% at month 3) given no grade ≥ 3 AEs at the time of dose escalation/all prior grade ≥ 3 AEs resolved to grade 1/2, and grade 2 non-hematologic toxicities resolved to grade ≤ 1. The protocol allowed dose reduction to bosutinib 300 mg QD for toxicity; following sponsor approval, further dose reduction to bosutinib 200 mg QD was permitted (for ≤ 4 weeks).

Treatment with bosutinib continued for approximately 3 years after registration of the last patient, representing the 12-month core treatment phase and the following ≥ 2-year extension phase, or until the end of the study, treatment failure, unacceptable toxicity, death, or withdrawal of consent.

### Study assessments

Molecular response was assessed at a central laboratory by quantitative reverse transcription polymerase chain reaction (RT-qPCR) using peripheral blood collected at baseline, every 3 months for the first 2 years of treatment, and then every 6 months thereafter on the international scale (IS) [12]. Cytogenetic response was locally assessed in Ph + patients using bone marrow aspirate obtained at baseline, every 3 months for the first 2 years of treatment, and every 6 months subsequently until complete cytogenetic response (CCyR) or MMR was achieved, after which bone marrow aspirates were only performed if clinically indicated. Hematologic response was assessed using peripheral blood collected locally, and by clinical evaluation of extramedullary disease. Mutation analysis was performed on central laboratory blood samples collected for RT-qPCR in the case of a lack of response, suboptimal response, or loss of response, and in patients who discontinued treatment.

Progression to AP-CML was defined as 15%–29% blasts in blood or marrow, > 30% blasts and promyelocytes in blood or marrow with blasts < 30%, or ≥ 20% basophils in blood. Progression to BP-CML was defined as ≥ 30% blasts in blood or bone marrow or extramedullary blast proliferation, other than in the spleen. Event-free survival (EFS) was defined as the time from the first dose of bosutinib to the occurrence of the earliest of the following events while on treatment: death due to any cause; transformation to AP/BP; loss of a complete hematologic response (CHR); loss of CCyR; and doubling of white blood cell count (≥ 1 month apart with a second value of > 20 × 10^9^/L and maintained in subsequent assessments for ≥ 2 weeks) in patients not achieving CHR. Overall survival (OS) was defined as the time from first dose of bosutinib to death due to any cause.

AEs were monitored throughout the study, coded using Medical Dictionary for Regulatory Activities (MedDRA) version 23.1, and severity was graded by the National Cancer Institute Common Terminology Criteria version 4.03 [13]. Treatment-emergent AEs (TEAEs) were defined as AEs that first occurred or worsened in severity after the first administration of bosutinib through 28 days after the last dose. For TEAE analysis, the following clustered terms were used for cytopenias: lymphopenia (Preferred Term [PT] = lymphopenia; lymphocyte count decreased), thrombocytopenia (PT = thrombocytopenia; platelet count decreased), anemia (PT = anemia; hemoglobin decreased), neutropenia (PT = neutropenia; neutrophil count decreased), and leukopenia (PT = leukopenia; white blood cell count decreased). The frequency and characteristics of TEAEs of special interest were analyzed by TEAE clusters, shown in Supplementary Table S1. TEAEs of special interest were analyzed by selecting MedDRA High Level Group Terms, PTs, and standardized MedDRA queries to generate TEAE clusters.

### Statistical analysis

The primary analysis population for efficacy evaluations was the modified as-treated population. The modified as-treated population consisted of all patients with Ph + CP-CML harboring b2a2/b3a2 transcripts who received ≥ 1 dose of bosutinib. Safety was analyzed in the as-treated population. The as-treated population included all patients who received ≥ 1 dose of bosutinib. Continuous variables were summarized using descriptive statistics, and categorical variables were summarized using frequencies and percentages.

MMR (≤ 0.1% *BCR*::*ABL1* on the IS, corresponding to ≥ 3-log reduction from standardized baseline with ≥ 3000 *ABL1* transcripts assessed) at month 12 in the modified as-treated population was the primary endpoint, as previously reported [11]. Secondary endpoints included MMR and CCyR by month 12, as previously reported [11], MMR by month 18, duration of MMR and CCyR, EFS, OS, and safety. Other efficacy assessments included cumulative MMR; molecular response^4^ (MR^4^) (≤ 0.01% *BCR*::*ABL1* on the IS, corresponding to ≥ 4-log reduction from standardized baseline with ≥ 9800 *ABL1* transcripts assessed); MR^4.5^ (≤ 0.0032% *BCR*::*ABL1* on the IS, corresponding to ≥ 4.5-log reduction from standardized baseline with ≥ 30,990 *ABL* transcripts assessed); and CCyR (0 Ph + chromosomes of ≥ 20 metaphases or MMR; bone marrow aspirates not required once MMR was achieved) throughout the study; time to MMR, MR^4^, MR^4.5^, and CCyR; time to transformation to AP- or BP-CML; and types of *BCR*::*ABL1* mutations present at treatment completion or discontinuation. Safety assessments included collection of AEs, serious AEs, vital signs, physical examinations, and laboratory assessments. Subgroup analyses of cumulative rates of molecular response and TEAEs were performed by Sokal score at screening, age, and modified Charlson comorbidity index (mCCI) score [14], which was calculated without the age component.

This final analysis is based on April 6, 2021, database lock (according to the protocol), after a minimum of 3 years of follow-up of the last enrolled patient.

## Results

### Patients and treatment

A total of 60 Japanese patients with newly diagnosed CP-CML were enrolled in the study and treated with bosutinib (Table 1, the as-treated population). All patients were Ph + and harbored b2a2/b3a2 transcripts, and were included in the modified as-treated population analyzed for efficacy. The as-treated population was identical with the modified as-treated population. Among the 60 patients, 46.7%, 41.7%, and 11.7% had low-, intermediate-, and high-risk Sokal scores, respectively, while 70.0% of patients had an mCCI score ≤ 2. The median (range) age was 55.0 (20–83 years); 31.7% of patients were aged ≥ 65 years. For other patient demographics and baseline clinical characteristics, please refer to the previously reported primary results [11].

**Table 1:** Patient demographics and baseline clinical characteristics used for subgroup analysis

	Bosutinib (*N* = 60)
Age, median (range), years	55 (20–83)
Age group, *n* (%)	
< 65 years	41 (68.3)
≥ 65 years	19 (31.7)
Sokal risk group, *n* (%)	
Low (< 0.8)	28 (46.7)
Intermediate (0.8–1.2)	25 (41.7)
High (> 1.2)	7 (11.7)
Modified Charlson comorbidity index, *n* (%)	
≤ 2	42 (70.0)
> 2	18 (30.0)

As-treated population. For other patient demographics and baseline clinical characteristics, refer to the previously reported primary results [11]. The proportions of patients with low- and intermediate-risk Sokal score were updated from those reported in the primary results [11] due to corrections added after the primary analysis

**Fig. 1:**
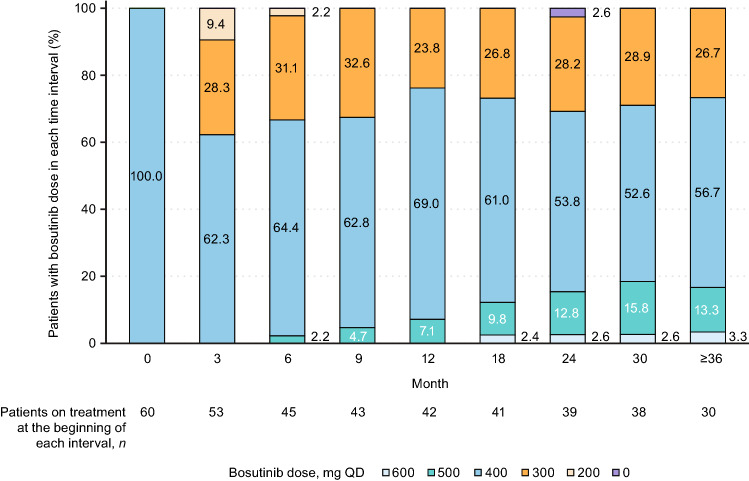
Bosutinib daily dose over time. As-treated population. Patients on treatment at each timepoint were included. The first non-zero actual dose in each interval was used; if all doses in each interval were 0 mg, they were counted as 0 mg. *QD* once daily

The median duration of treatment was 35.9 months (range 0.3–44.2 months; Table 2), and the median duration of follow-up was 39.2 months (range 13.2–45.1 months). The median dose intensity over the 3-year period was 357.4 mg/day (range 95.3–548.1 mg/day). Among the patients on treatment, approximately 70% were dosed at 400 mg or higher at each timepoint across the treatment period (Fig. 1).

**Table 2:** Treatment summary

	Bosutinib (*N* = 60)
Duration of follow-up, median (range), months	39.2 (13.2–45.1)
Duration of treatment, median (range), months	35.9 (0.3–44.2)
Discontinued treatment, *n* (%)	24 (40.0)
Adverse event	21 (35.0)
Physician’s decision	1 (1.7)
Other^a^	2 (3.3)
Dose reduction due to adverse events, *n* (%)	36 (60.0)
400 to 300 mg QD	36 (60.0)
300 to 200 mg QD	9 (15.0)
Dose interruption due to adverse events, *n* (%)	50 (83.3)
Dose escalation due to unsatisfactory response,^b^ *n* (%)	10 (16.7)
400 to 500 mg QD	10 (16.7)
500 to 600 mg QD	1 (1.7)
Dose intensity, median (range), mg/day	357.4 (95.3–548.1)

As-treated population

*AE* adverse event, *QD* once daily

^a^Other reasons for treatment discontinuation were initiation of treatment for secondary malignancy, and inability to visit the study site due to a patient relocation

^b^*BCR*::*ABL1* transcripts > 10% and/or Philadelphia chromosome–positive metaphases > 35% at month 3 or month 6, or any time in the case of lack of response; no grade ≥ 3 AEs at the time of dose escalation; all prior grade ≥ 3 AEs resolved to grade ≤ 2 AEs; and all grade 2 non-hematologic toxicities resolved to grade ≤ 1

At study completion, 36 (60.0%) patients were still on treatment. Out of 24 (40.0%) patients who discontinued treatment, the most common reason for treatment discontinuation was AEs (35.0%). Subsequent TKI treatments after bosutinib were dasatinib (18.3%), nilotinib (15.0%), imatinib (11.7%), bosutinib (6.7%), and ponatinib (3.3%; Supplementary Table S6). Dose interruptions or reductions due to AEs occurred in 50 (83.3%) and 36 (60.0%) patients, respectively. The median time to first dose interruption was 20.0 days (range 3–1008 days), and the median duration of dose interruption was 34.5 days (range 1–136 days). A total of 36 patients (60.0%) had their dose reduced from 400 to 300 mg, and 9 patients (15.0%) had their dose further reduced from 300 to 200 mg. For the dose reductions to 300 mg, the median time to the first dose reduction was 55.0 days (range 4–1011 days) and median duration of dose reduction was 68.0 days (range 5–1081 days). For the dose reductions to 200 mg, the median time to the first dose reduction was 116.0 days (range 37–819 days), and the median duration of dose reduction was 26.0 days (range 8–36 days). Dose escalations to 500 mg QD due to unsatisfactory response occurred in 10 (16.7%) patients. One patient (1.7%) had a further dose escalation to 600 mg QD. The median time from the first dose of study drug to dose escalation to 500 mg was 419.0 days (range 99–1003 days).

### Efficacy

The MMR rate (90% CI) by month 18 was 66.7% (56.7–76.7). The cumulative rates (90% CI) of MMR, MR^4^, and MR^4.5^ at any time on treatment were 70.0% (60.3–79.7), 53.3% (42.7–63.9), and 48.3% (37.7–58.9), respectively (Table 3; Supplementary Fig. S1). Among patients who achieved MMR or MR^4^, none had a confirmed loss of response. Of patients with dose reductions to 300 (*n* = 36) and 200 (*n* = 9) mg QD, 63.9% (*n* = 23) and 33.3% (*n* = 3), respectively, achieved MMR or maintained a previously attained MMR after dose reduction (Supplementary Table S2). Among the patients with MMR achieved/maintained after dose reduction to 300 and 200 mg, 73.9% (*n* = 17) and 66.7% (*n* = 2) attained the first MMR following dose reduction, respectively. Meanwhile, among patients with dose escalation to 500 mg QD (*n* = 10), 90.0% (*n* = 9) achieved MMR or maintained a previously attained MMR after dose escalation (Supplementary Table S2). Among the patients with MMR achieved/maintained after dose escalation to 500 mg, 22.2% (*n* = 2) attained the first MMR following dose escalation. For the patient with dose escalation to 600 mg QD (*n* = 1), first MMR was achieved following dose escalation. The cumulative CCyR rate (90% CI) at any time was 80.0% (71.5–88.5), and among patients who achieved CCyR, none had a confirmed loss of response. Of responders, the median (range) time to CCyR, MMR, MR^4^, and MR^4.5^ was 12.2 (11.9–36.1), 24.1 (12.0–143.1), 42.1 (23.7–168.9), and 48.1 (23.9–177.3) weeks, respectively.

**Table 3:** Cumulative molecular response rates

*n* (%)	Bosutinib (*N* = 60)					
	By month 12	By month 18	By month 24	By month 30	By month 36	Any time on treatment
MMR	37 (61.7)	40 (66.7)	40 (66.7)	41 (68.3)	42 (70.0)	42 (70.0)
90% CI	51.3–72.0	56.7–76.7	56.7–76.7	58.5–78.2	60.3–79.7	60.3–79.7
MR^4^	20 (33.3)	25 (41.7)	28 (46.7)	29 (48.3)	30 (50.0)	32 (53.3)
90% CI	23.3–43.3	31.2–52.1	36.1–57.3	37.7–58.9	39.4–60.6	42.7–63.9
MR^4.5^	17 (28.3)	20 (33.3)	21 (35.0)	26 (43.3)	28 (46.7)	29 (48.3)
90% CI	18.8–37.9	23.3–43.3	24.9–45.1	32.8–53.9	36.1–57.3	37.7–58.9

Modified as-treated population. MMR =  ≤ 0.1% *BCR*::*ABL1* on the IS with ≥ 3000 *ABL1* transcripts assessed; MR^4^ =  ≤ 0.01% *BCR*::*ABL1* on the IS with ≥ 9800 *ABL1* transcripts assessed; MR^4.5^ =  ≤ 0.0032% *BCR*::*ABL1* on the IS with ≥ 30,990 *ABL1* transcripts assessed. 90% CIs based on the Brookmeyer–Crowley method

*IS* international scale, *MMR* major molecular response, *MR* molecular response

The cumulative incidence (90% CI) of EFS events at 3 years was 1.7% (0.2–6.4) (Table 4; Supplementary Fig. S2). The only EFS event occurring during the follow-up period was loss of CHR, which occurred in 1 patient. The 3-year Kaplan–Meier OS estimate (90% CI) was 96.7% (89.7–98.9) (Table 4; Supplementary Fig. S3). Two (3.3%) patients died on study after the safety reporting period (> 28 days after the last dose of bosutinib; Table 4). One (1.7%) patient discontinued bosutinib on day 85 due to an AE (thrombocytopenia) and died on day 403 due to disease progression. This patient was treated with dasatinib as a second-line treatment. One (1.7%) patient discontinued bosutinib on day 64 due to an AE (pneumonia) and died on day 730 due to a cerebral hemorrhage considered unrelated to bosutinib. This patient was treated with ponatinib followed by nilotinib as follow-up anti-cancer therapy. No patients progressed to AP-/BP-CML while on treatment (Table 4).

**Table 4:** Summary of transformations, event-free survival, and overall survival

	Bosutinib (*N* = 60)
Patients with on-treatment transformation to AP/BP, *n*	0
Patients with EFS events, *n* (%)	1 (1.7)
Cumulative incidence of progression or death at month 36, % (90% CI)^a^	1.7 (0.2–6.4)
Number of deaths, *n* (%)	2 (3.3)
Kaplan–Meier OS at month 36, % (90% CI)^b^	96.7 (89.7–98.9)

Modified as-treated population

*AP* accelerated phase, *BP* blast phase, *EFS* event-free survival, *OS* overall survival

^a^Based on the delta method with the log(-log) transformation

^b^Based on the Greenwood’s log(-log) transformation

Of the 24 patients who had mutation testing while on treatment or at treatment completion, 21 were evaluable; no patients had an emergent mutation.

#### MMR by subgroup

Cumulative rates of MMR (90% CI) at any time in patients with low-, intermediate-, and high-risk Sokal scores were 57.1% (41.8–72.5), 88.0% (77.3–98.7), and 57.1% (26.4–87.9), respectively (Supplementary Table S3; Supplementary Fig. S4). Cumulative rates of MMR (90% CI) at any time in patients < 65 years and ≥ 65 years were 73.2% (61.8–84.6) and 63.2% (45.0–81.4), respectively (Supplementary Table S3). Cumulative rates of MMR (90% CI) at any time in patients with mCCI scores of ≤ 2 or > 2 were 69.0% (57.3–80.8) and 72.2% (54.9–89.6), respectively (Supplementary Table S3).

### Safety

**Fig. 2:**
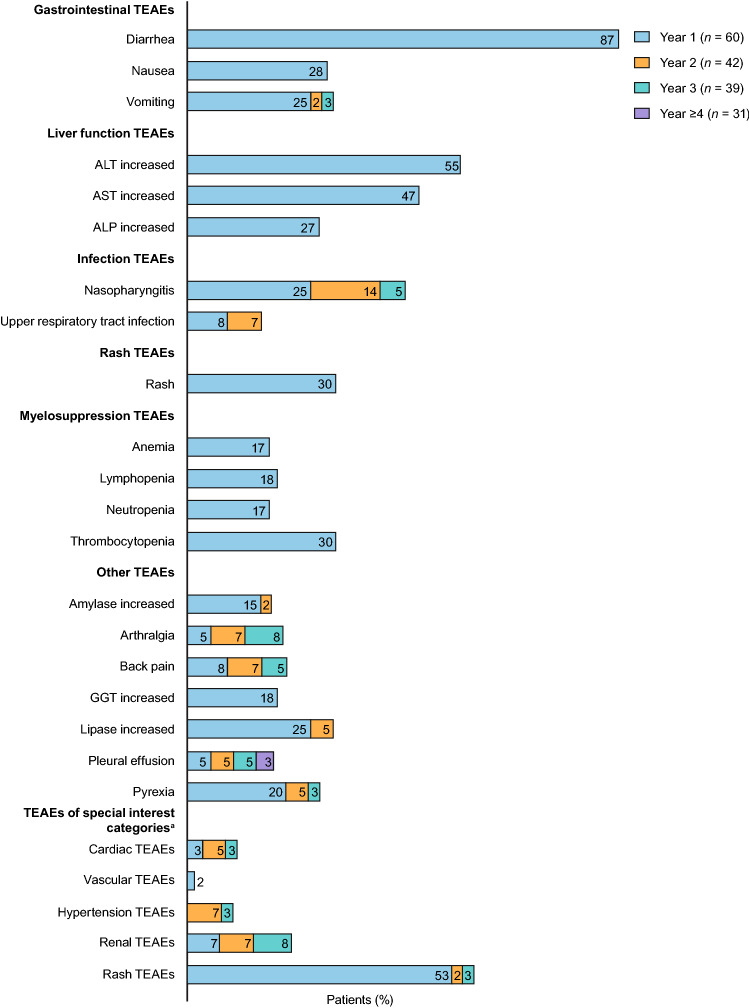
Cumulative incidence (≥ 15%)^a^ of newly occurring TEAEs of any grade, by treatment year. As-treated population. One year = 365.25 days. ^a^Definitions of TEAEs of special interest categories (cardiac, vascular, hypertension, renal, rash) are provided in Supplementary Table S1. *ALP* alkaline phosphatase; *ALT* alanine aminotransferase, *AST* aspartate aminotransferase, *GGT* gamma-glutamyltransferase, *TEAE* treatment-emergent adverse event

The incidence of all-causality, any grade TEAEs was 100% and the incidence of serious TEAEs was 25.0% (Supplementary Table S4). The most common TEAEs are shown in Table 5. The 3 most common all-causality, any grade TEAEs were diarrhea (86.7%), increased alanine aminotransferase (ALT; 55.0%), and increased aspartate aminotransferase (AST; 46.7%). The cumulative incidence of newly occurring TEAEs by year is shown in Fig. 2; most occurred within the first year of treatment. The most common (≥ 5%) categories of newly observed TEAEs in year 2 or later in patients who were on treatment at year 2 (*n* = 42) were infection (23.8%), effusion (14.3%), renal (14.3%), hypertension (9.5%), and cardiac (7.1%). The most common individual TEAEs of these categories were nasopharyngitis (19.0%), pleural effusion (11.9%), blood creatinine increased (11.9%), hypertension (9.5%), and cardiac failure (4.8%), respectively. There were no newly observed TEAEs in year 2 or later in the myelosuppression and vascular TEAE categories. Grade ≥ 3 TEAEs occurred in 81.7% of patients (Table 5). Grade ≥ 3 TEAEs reported in ≥ 10% of patients were increased ALT (33.3%), increased AST (18.3%), increased lipase (16.7%), diarrhea (15.0%), lymphopenia (13.3%), and neutropenia (11.7%).

**Table 5:** Treatment-emergent adverse events^a^

TEAE cluster, *n* (%) MedDRA PT, *n* (%)	Bosutinib (*N* = 60)			
	All-causality		Treatment-related	
	Any grade	Grade ≥ 3	Any grade	Grade ≥ 3
Any TEAE	60 (100)	49 (81.7)	60 (100)	47 (78.3)
Gastrointestinal TEAEs	52 (86.7)	10 (16.7)	52 (86.7)	10 (16.7)
Diarrhea	52 (86.7)	9 (15.0)	52 (86.7)	9 (15.0)
Nausea	17 (28.3)	0	16 (26.7)	0
Vomiting	17 (28.3)	2 (3.3)	14 (23.3)	2 (3.3)
Liver function TEAEs	48 (80.0)	30 (50.0)	47 (78.3)	29 (48.3)
ALT increased	33 (55.0)	20 (33.3)	33 (55.0)	20 (33.3)
AST increased	28 (46.7)	11 (18.3)	28 (46.7)	11 (18.3)
Blood ALP increased	16 (26.7)	0	16 (26.7)	0
Liver disorder	7 (11.7)	5 (8.3)	6 (10.0)	5 (8.3)
Infection TEAEs	45 (75.0)	4 (6.7)	8 (13.3)	4 (6.7)
Nasopharyngitis	23 (38.3)	0	2 (3.3)	0
Upper respiratory tract infection	8 (13.3)	0	1 (1.7)	0
Influenza	7 (11.7)	1 (1.7)	0	0
Gastroenteritis	6 (10.0)	1 (1.7)	1 (1.7)	1 (1.7)
Rash TEAEs	34 (56.7)	3 (5.0)	29 (48.3)	3 (5.0)
Rash	18 (30.0)	1 (1.7)	15 (25.0)	1 (1.7)
Maculo-papular rash	8 (13.3)	1 (1.7)	8 (13.3)	1 (1.7)
Myelosuppression TEAEs	27 (45.0)	17 (28.3)	27 (45.0)	17 (28.3)
Thrombocytopenia	18 (30.0)	5 (8.3)	18 (30.0)	5 (8.3)
Lymphopenia	11 (18.3)	8 (13.3)	11 (18.3)	8 (13.3)
Anemia	10 (16.7)	1 (1.7)	10 (16.7)	1 (1.7)
Neutropenia	10 (16.7)	7 (11.7)	10 (16.7)	7 (11.7)
Leukopenia	6 (10.0)	2 (3.3)	6 (10.0)	2 (3.3)
Renal TEAEs	10 (16.7)	1 (1.7)	9 (15.0)	1 (1.7)
Increased blood creatinine	6 (10.0)	0	5 (8.3)	0
Cardiac TEAEs	5 (8.3)	0	5 (8.3)	0
Hypertension TEAEs	4 (6.7)	1 (1.7)	3 (5.0)	1 (1.7)
Vascular TEAEs	1 (1.7)	0	0	0
Other TEAEs				
Increased lipase	17 (28.3)	10 (16.7)	16 (26.7)	9 (15.0)
Pyrexia	15 (25.0)	1 (1.7)	8 (13.3)	1 (1.7)
Increased GGT	11 (18.3)	3 (5.0)	11 (18.3)	3 (5.0)
Back pain	10 (16.7)	0	1 (1.7)	0
Increased amylase	10 (16.7)	1 (1.7)	9 (15.0)	1 (1.7)
Arthralgia	9 (15.0)	0	2 (3.3)	0
Constipation	8 (13.3)	0	5 (8.3)	0
Pleural effusion	8 (13.3)	1 (1.7)	8 (13.3)	1 (1.7)
Headache	7 (11.7)	0	5 (8.3)	0
Increased blood creatinine phosphokinase	6 (10.0)	2 (3.3)	3 (5.0)	1 (1.7)
Upper abdominal pain	6 (10.0)	0	5 (8.3)	0
Stomatitis	6 (10.0)	0	1 (1.7)	0

As-treated population. ^a^Any grade TEAEs reported in ≥ 10% of patients and TEAE clusters of interest (cardiac, hypertension, and vascular TEAEs), including those with < 10% frequency. TEAEs occurring in ≥ 10% of patients that do not belong to any TEAE cluster above are summarized in the “other” category; coded using MedDRA version 23.1 PTs and graded according to CTCAE version 4.03

*ALP* alkaline phosphatase, *ALT* alanine aminotransferase, *AST* aspartate aminotransferase, *CTCAE* Common Terminology Criteria for Adverse Events, *GGT* gamma-glutamyltransferase, *MedDRA* Medical Dictionary for Regulatory Activities, *PT* Preferred Term, *TEAE* treatment-emergent adverse event

Serious AEs (all-causality) occurred in 25.0% of patients. Serious AEs observed in 2 or more patients were diarrhea, dehydration, liver disorder, and pneumonia (2 patients [3.3%] each). There were no deaths on treatment (< 28 days after the last dose of bosutinib).

TEAEs led to bosutinib dose interruption or dose reduction in 73.3% and 61.7% of patients, respectively. The most common TEAEs (≥ 10% of patients) leading to dose interruption were increased ALT (28.3%), increased AST (16.7%), diarrhea (11.7%), and liver disorder (10.0%). The most common TEAEs (≥ 10% of patients) leading to dose reduction were increased ALT (21.7%) and increased AST (13.3%). Among 24 (40.0%) of patients who discontinued treatment, the most common reason for treatment discontinuation was AEs (35.0%). TEAEs leading to treatment discontinuation in ≥ 2% of patients were increased ALT (10.0%), increased AST (8.3%), increased lipase (3.3%), drug eruption (3.3%), and erythema multiforme (3.3%) (Supplementary Table S5). TEAEs in the liver category were the most common type of TEAEs leading to treatment discontinuation, involving 10 (16.7%) patients. Other TEAEs leading to treatment discontinuation included neutropenia, thrombocytopenia, pleural effusion, toxic nephropathy, increased blood creatinine, increased pancreatic enzymes, increased hepatic enzymes, drug-induced liver injury, liver disorder, and breast cancer (1.7% each). Among the patients with TEAEs leading to treatment discontinuation (*n* = 21), the majority of TEAEs leading to discontinuation occurred within the first year of treatment (81.0%), especially in the first 6 months (71.4%), and were less frequent thereafter. Of the 10 patients who discontinued bosutinib due to liver TEAEs, 5 patients, 3 patients, and 2 patients were treated with dasatinib, imatinib, and nilotinib, respectively, as the second-line treatment.

Analyzed by age and mCCI score, 100% of patients experienced any grade TEAEs; 24.4% and 26.3% of patients aged < 65 years and ≥ 65 years experienced serious TEAEs, respectively, while 28.6% and 16.7% of patients with an mCCI score ≤ 2 and > 2 experienced serious TEAEs, respectively (Supplementary Table S4). Grade ≥ 3 TEAEs were observed in 75.6% and 94.7% of patients aged < 65 years and ≥ 65 years, respectively, and 81.0% and 83.3% of patients with an mCCI score of ≤ 2 or > 2, respectively. The most common TEAEs (any grade and grade ≥ 3) by age and mCCI are shown in Supplementary Fig. S5. The most common TEAEs (any grade) for patients aged < 65 years, ≥ 65 years, and with an mCCI score ≤ 2, respectively, were diarrhea (90.2%, 78.9%, 95.2%), ALT increased (53.7%, 57.9%, 59.5%), and AST increased (46.3%, 47.4%, 52.4%). For patients with an mCCI score > 2, the most common TEAEs (any grade) were diarrhea (66.7%), ALT increased (44.4%), and nasopharyngitis (38.9%). TEAEs leading to dose modification were frequently observed in patients aged ≥ 65 years versus < 65 years. TEAEs leading to discontinuation, dose reduction, and temporarily interruption were observed in 57.9% versus 24.4%, 78.9% versus 53.7%, and 94.7% versus 63.4% of patients, respectively. The higher dose modification was reflected in lower median dose intensity of bosutinib in patients aged ≥ 65 years versus < 65 years (292.1 mg/day vs 384.0 mg/day). As previously reported, bosutinib trough concentrations were similar between patients aged ≥ 65 years and < 65 years [11].

When evaluated according to categories of special interest (Supplementary Table S1), the most frequently reported TEAE categories of any grade were gastrointestinal (86.7%), liver function (80.0%), rash (56.7%), and myelosuppression (45.0%) (Supplementary Table S7). The most common grade ≥ 3 TEAE categories of special interest were liver function (50.0%), myelosuppression (28.3%), and gastrointestinal (16.7%). Of the 52 (86.7%) patients with gastrointestinal TEAEs, all TEAEs were considered to be related to bosutinib. The maximum toxicity was grade 1 in 28.3% of patients, grade 2 in 41.7%, and grade 3 in 16.7%. No patients discontinued bosutinib due to a gastrointestinal TEAE. In addition, 8 (15.4%) patients temporarily stopped bosutinib because of gastrointestinal TEAEs; all patients were successfully rechallenged. Of the 48 (80.0%) patients with liver function TEAEs, 47 (78.3%) had events considered bosutinib-related. The maximum toxicity was grade 1 in 16.7% of patients, grade 2 in 13.3%, grade 3 in 41.7%, and grade 4 in 8.3%. Ten (16.7%) patients permanently discontinued bosutinib due to liver function TEAEs. In addition, 29 (60.4%) patients temporarily stopped bosutinib because of liver function TEAEs; 20 (71.4%) were successfully rechallenged. The median duration of temporary treatment stop was 18.5 days (range: 8–48). Of the 34 (56.7%) patients with rash TEAEs, 29 (48.3%) had events considered bosutinib-related. The maximum toxicity was grade 1 in 30.0% of patients, grade 2 in 21.7%, and grade 3 in 5.0%. No patients discontinued bosutinib due to a rash TEAE. In addition, 4 (11.8%) patients temporarily stopped bosutinib because of rash TEAEs; all patients were successfully rechallenged. Of the 27 (45.0%) patients with myelosuppression TEAEs, all TEAEs were considered related to bosutinib. The maximum toxicity was grade 1 in 8.3% of patients, grade 2 in 8.3%, grade 3 in 26.7%, and grade 4 in 1.7%. Two (3.3%) patients permanently discontinued bosutinib due to myelosuppression TEAEs. In addition, 10 (37.0%) patients temporarily stopped bosutinib because of myelosuppression TEAEs; all patients were successfully rechallenged.

The incidence of any grade cardiac, vascular, hypertension, and renal TEAEs was 8.3%, 1.7%, 6.7%, and 16.7%, respectively (Supplementary Table S8). Cardiac TEAEs included pericardial effusion (5.0%) and cardiac failure (3.3%), all of which were grade ≤ 2. Among the 5 patients with cardiac TEAEs, 1 patient with pericardial effusion had a history of diabetes mellitus, hyperlipidaemia, and hypertension, and another patient with cardiac failure had a history of hypertension, which are known risk factors of cardiovascular events during TKI treatment [15]. The only reported vascular TEAE was grade 1 peripheral coldness for 1 (1.7%) patient who had a history of diabetes mellitus. The only hypertension TEAE reported was hypertension (6.7%); only 1 patient (1.7%) experienced grade ≥ 3 hypertension, and 2 patients (3.3%) had a history of hypertension (Supplementary Table S7). Renal TEAEs (all-causality) included blood creatinine increase (10.0%), acute kidney injury (3.3%), chronic kidney disease, renal impairment, and renal injury (all 1.7%); only 1 patient (1.7%) experienced a grade ≥ 3 TEAE (chronic kidney disease), which was not considered treatment-related, and only 1 patient (1.7%) had a history of the same TEAE category (Supplementary Table S7).

## Discussion

In Japanese patients with newly diagnosed CP-CML, bosutinib met the primary objective of this phase 2 study (MMR at month 12) [11]. After a minimum 3-year follow-up, a clinically meaningful benefit was maintained over time with high response rates. The data reported further suggest that bosutinib is an effective first-line treatment option for Japanese patients with newly diagnosed CP-CML, in addition to second- or later lines of therapy in Japan.

Approximately half of the patients treated with bosutinib achieved deep MR during the study. The responses were durable as no loss of molecular responses was observed. These are clinically meaningful endpoints in CML since they are associated with a lower risk of disease progression and with improved EFS, progression-free survival, and OS. Consistent with the response rates presented, there was no on-treatment progression to AP- or BP-CML. In addition, few on-treatment EFS events and on-study deaths were observed, which was also expected from a first-line CP-CML population. The achievement of a rapid, deep, and durable MR may also allow the eventual discontinuation of TKI therapy [16]. The efficacy results of this study suggest the potential for a beneficial long-term outcome for patients treated with bosutinib, being in alignment with the treatment goal of achieving treatment-free remission for patients with newly diagnosed CP-CML [17]. Compared with the bosutinib arm of the global phase 3 BFORE trial of first-line bosutinib [4, 18], more patients experienced dose reduction and/or interruption, which led to lower dose intensity. However, the overall efficacy results were consistent with findings from the BFORE trial, which established improved efficacy of bosutinib versus imatinib in patients with newly diagnosed CP-CML [4, 18]. Subgroup analysis by Sokal score, age, mCCI score, and *BCR*::*ABL1* transcripts at 3 months (early MR) was conducted for response rates and time to response. Overall, favorable response rates were maintained across all subgroups. The achievement of *BCR*::*ABL1* transcript levels ≤ 10% at 3 months and low-risk Sokal score was known to be related to better prognosis [19–21], but in this subgroup analysis, no clear relationship was detected between *BCR*::*ABL1* transcripts at 3 months/Sokal risk and response rates. However, careful interpretation is required since the number of patients in some subgroups was small, especially for that of patients not achieving *BCR*::*ABL1* transcript levels ≤ 10% at 3 months. In this study, CCyR, MMR, and MR^4.5^ by 3 years was 80.0%, 70.0%, and 46.7%, respectively. These results were similar to those reported from a phase 3 study of nilotinib and dasatinib in patients with newly diagnosed CML in Japan [22]. In that study, the CCyR, MMR, and MR^4.5^ by 3 years was 78.4%, 73.1%, and 40.5% for nilotinib; and 78.9%, 77.1%, and 44.5% for dasatinib. This suggests bosutinib has comparable efficacy to nilotinib and dasatinib for the treatment of Japanese patients with newly diagnosed CP-CML.

The safety profile of bosutinib in this study was consistent with what has been described previously in the Japanese 12-month primary analysis [11], with no new safety signals identified after long-term follow-up. There were some TEAEs with increased incidence after longer follow-up, including cardiac, effusion, hypertension, infection, and renal TEAEs (Fig. 2). However, these TEAEs are not commonly observed with bosutinib. The most common individual TEAEs with bosutinib are mainly gastrointestinal, liver function, and rash TEAEs, which was consistent with the global BFORE trial [4, 18] and previously reported data in Japanese patients [10]. The rate of grade ≥ 3 TEAEs in bosutinib-treated patients was higher in the Japanese population than in the BFORE population (81.7% vs 73.5%) [18]. However, the rates of any grade TEAEs (100% vs 98.9%) were similar across the 2 studies, and serious TEAEs were slightly lower in the Japanese population than in BFORE (25.0% vs 36.6%). A greater proportion of patients in this study discontinued bosutinib due to TEAEs when compared with the BFORE population (35.0% vs 25.4%) [18]. The most common TEAEs leading to discontinuation of bosutinib in this study when compared with the BFORE trial were increased ALT (10.0% vs 4.9%) and increased AST (8.3% vs 2.6%) [18]. Moreover, a greater proportion of patients in this study discontinued bosutinib due to TEAEs compared with patients treated with dasatinib (35.0% vs 19.0%) and nilotinib 300 and 400 mg twice daily (35.0% vs 10.3% and 13.0%, respectively) [23, 24]. However, most of the TEAEs leading to treatment discontinuation occurred within the first 6 months, and the majority of patients who were on treatment at month 6 were able to continue bosutinib treatment thereafter. Therefore, it is considered important to closely monitor patients during the initial phase of bosutinib treatment. Most TEAEs associated with bosutinib treatment were manageable with dose reductions/dose interruptions or supportive treatment. Subgroup analyses revealed some differences in incidence of TEAEs according to age and mCCI score, particularly in more frequently observed TEAEs leading to dose modification in patients aged ≥ 65 years versus < 65 years; however, the kind and frequency of major TEAEs were generally similar.

An analysis of the TEAEs of special interest did not identify any new safety signals with longer follow-up (at least 3 years) and the majority of TEAEs occurred within 12 months and were reported in the primary analysis [11]. While gastrointestinal TEAEs, including diarrhea and vomiting, occurred more frequently in the study compared with the bosutinib arm of the BFORE trial [18], they occurred early on treatment, were generally of short duration, and did not lead to treatment discontinuation. Liver function TEAEs were also frequently observed in this study. This trend of higher incidence in Japanese patients with CML was also confirmed in previous bosutinib studies [9, 10], and therefore more frequent monitoring of liver function (i.e., every 1–2 weeks during the first few months of treatment) is recommended in the prescribing information in Japan [2], compared with the prescribing information in the United States [1]. Even though this type of AE, including increased ALT and AST, was the most frequent cause of permanent treatment discontinuation, the majority of patients with liver function events were successfully rechallenged and were able to remain on long-term treatment. This is consistent with previous observations, with liver function TEAEs often improving or resolving with time and dose-management strategies [25]. In addition to gastrointestinal and liver function TEAEs, several TEAEs (with ≥ 5% difference) in TEAEs of special interest categories were observed more frequently in this study compared with the bosutinib arm of BFORE [18]. These included pleural effusion under the effusion category, nasopharyngitis under the infection category, and rash and rash maculo-papular under the rash category. All collective TEAEs of special interest under the renal category were also frequently observed (with ≥ 5% difference) in this study compared with the bosutinib arm of BFORE [18]. The incidence of treatment discontinuation due to these TEAEs was low. Most of the events in the effusion, infection, and renal categories were grade 1 or 2, and considered manageable/reversible. The majority of the infection events were judged as not related to bosutinib treatment. The rate of pleural effusion in this study (13.3%) appears to be lower than what has been previously reported in patients receiving first-line dasatinib after 5 years (28.0%; 42.0% in the subset of Japanese patients) [23, 26]. TEAEs of special interest of nausea under the gastrointestinal category, urinary tract infection under the infection category, and the categories of edema, hemorrhage, and vascular events were observed more frequently (with ≥ 5% difference) in the bosutinib arm of BFORE [18] compared with this study. In this study, all the events in the edema, hemorrhage, and vascular categories were grade 1 or 2, and no patient discontinued due to these TEAEs in this study. In addition to vascular events, all the cardiac events were grade 1 or 2, none of which led to treatment discontinuation. Cardiovascular health is evolving as an important consideration in patients with CML, not only because of the improved prognosis being enjoyed by patients treated with TKI-based therapies, but also because some later-generation *BCR*::*ABL1* TKIs have been associated with cardiovascular complications [27]. These issues have heightened the need for cardiovascular risk assessment in patients with CML being initiated on TKI therapy, and a recently published consensus document has provided important governance on risk assessment, stratification, treatment, and monitoring [28].

The study has a number of limitations. These include the small number of patients, especially in the subgroup analyses, and a single-arm design with no comparator. However, the longer-term follow-up compared with the primary analysis confirms that deep MR with bosutinib is maintained in Japanese patients with newly diagnosed CP-CML. Furthermore, in the safety analysis, data from this study are described in comparison with BFORE trial data [18]; however, caution should be exercised for the interpretation because the follow-up duration was different for the 2 studies (5 years of follow-up for BFORE). In addition, this analysis enrolled a higher proportion of patients with a low-risk Sokal score and an ECOG PS score of 0 than the BFORE study, suggesting that close management of AEs might be beneficial in real-world clinical practice where patients may have a broader range of prognostic risk.

In conclusion, based on the overall efficacy and safety data, bosutinib continues to demonstrate a favorable benefit/risk profile and is an important treatment option for Japanese patients with newly diagnosed CP-CML. Optimal management of TEAEs during early treatment with bosutinib should be prioritized.

## Supplementary Information

Below is the link to the electronic supplementary material.

Supplementary file1 (PDF 4896 KB)

## Data Availability

Upon request, and subject to review, Pfizer will provide the data that support the findings of this study. Subject to certain criteria, conditions and exceptions, Pfizer may also provide access to the related individual de-identified participant data. See https://www.pfizer.com/science/clinical-trials/trial-data-and-results for more information.

## References

[1] Pfizer Inc. Bosulif® (bosutinib) prescribing information. US Food and Drug Administration, New York, NY. 2012. https://www.accessdata.fda.gov/drugsatfda_docs/label/2017/203341s009lbl.pdf. Accessed 11 Nov 2019.

[2] Pfizer Japan Inc. Bosulif® (bosutinib hydrate) prescribing information. Japan2020.

[3] European Medicines Agency. Bosulif® (bosutinib) summary of product characteristics. European Medicines Agency, London. 2013. https://www.ema.europa.eu/en/medicines/human/EPAR/bosulif. Accessed 11 Nov 2019.

[4] Cortes JE, Gambacorti-Passerini C, Deininger MW, Mauro MJ, Chuah C, Kim DW (2018). Bosutinib versus imatinib for newly diagnosed chronic myeloid leukemia: results from the randomized BFORE trial. J Clin Oncol.

[5] Cortes JE, Kim DW, Kantarjian HM, Brümmendorf TH, Dyagil I, Griskevicius L (2012). Bosutinib versus imatinib in newly diagnosed chronic-phase chronic myeloid leukemia: results from the BELA trial. J Clin Oncol.

[6] Cortes JE, Kantarjian HM, Brümmendorf TH, Kim DW, Turkina AG, Shen ZX (2011). Safety and efficacy of bosutinib (SKI-606) in chronic phase Philadelphia chromosome-positive chronic myeloid leukemia patients with resistance or intolerance to imatinib. Blood.

[7] Gambacorti-Passerini C, Brümmendorf TH, Kim DW, Turkina AG, Masszi T, Assouline S (2014). Bosutinib efficacy and safety in chronic phase chronic myeloid leukemia after imatinib resistance or intolerance: minimum 24-month follow-up. Am J Hematol.

[8] Gambacorti-Passerini C, Cortes JE, Lipton JH, Kantarjian HM, Kim DW, Schafhausen P (2018). Safety and efficacy of second-line bosutinib for chronic phase chronic myeloid leukemia over a five-year period: final results of a phase I/II study. Haematologica.

[9] Nakaseko C, Takahashi N, Ishizawa K, Kobayashi Y, Ohashi K, Nakagawa Y (2015). A phase 1/2 study of bosutinib in Japanese adults with Philadelphia chromosome-positive chronic myeloid leukemia. Int J Hematol.

[10] Takahashi N, Nakaseko C, Kobayashi Y, Miyamura K, Ono C, Koide Y (2017). Long-term treatment with bosutinib in a phase 1/2 study in Japanese chronic myeloid leukemia patients resistant/intolerant to prior tyrosine kinase inhibitor treatment. Int J Hematol.

[11] Hino M, Matsumura I, Fujisawa S, Ishizawa K, Ono T, Sakaida E (2020). Phase 2 study of bosutinib in Japanese patients with newly diagnosed chronic phase chronic myeloid leukemia. Int J Hematol.

[12] Hughes T, Deininger M, Hochhaus A, Branford S, Radich J, Kaeda J (2006). Monitoring CML patients responding to treatment with tyrosine kinase inhibitors: review and recommendations for harmonizing current methodology for detecting BCR-ABL transcripts and kinase domain mutations and for expressing results. Blood.

[13] National Cancer Institute. Common Terminology Criteria for Adverse Events (CTCAE) v4.03. US Department of Health and Human Services (2010) https://evs.nci.nih.gov/ftp1/CTCAE/CTCAE_4.03/CTCAE_4.03_2010-06-14_QuickReference_8.5x11.pdf. Accessed 11 Nov 2019.

[14] Charlson ME, Pompei P, Ales KL, MacKenzie CR (1987). A new method of classifying prognostic comorbidity in longitudinal studies: development and validation. J Chronic Dis.

[15] Rea D, Mirault T, Raffoux E, Boissel N, Andreoli AL, Rousselot P (2015). Usefulness of the 2012 European CVD risk assessment model to identify patients at high risk of cardiovascular events during nilotinib therapy in chronic myeloid leukemia. Leukemia.

[16] Annunziata M, Bonifacio M, Breccia M, Castagnetti F, Gozzini A, Iurlo A (2020). Current strategies and future directions to achieve deep molecular response and treatment-free remission in chronic myeloid leukemia. Front Oncol.

[17] Hochhaus A, Baccarani M, Silver RT, Schiffer C, Apperley JF, Cervantes F (2020). European LeukemiaNet 2020 recommendations for treating chronic myeloid leukemia. Leukemia.

[18] Brümmendorf TH, Cortes JE, Milojkovic D, Gambacorti-Passerini C, Clark RE, le Coutre P (2022). Bosutinib versus imatinib for newly diagnosed chronic phase chronic myeloid leukemia: final results from the BFORE trial. Leukemia.

[19] Hanfstein B, Müller M, Hehlmann R, Erben P, Lauseker M, Fabarius A (2012). Early molecular and cytogenetic response is predictive for long-term progression-free and overall survival in chronic myeloid leukemia (CML). Leukemia.

[20] Baccarani M, Deininger MW, Rosti G, Hochhaus A, Soverini S, Apperley JF (2013). European LeukemiaNet recommendations for the management of chronic myeloid leukemia: 2013. Blood.

[21] Marin D, Ibrahim AR, Lucas C, Gerrard G, Wang L, Szydlo RM (2012). Assessment of BCR-ABL1 transcript levels at 3 months is the only requirement for predicting outcome for patients with chronic myeloid leukemia treated with tyrosine kinase inhibitors. J Clin Oncol.

[22] Matsumura I, Ohtake S, Atsuta Y, Kurata M, Minami Y, Takahashi N (2020). Nilotinib vs. dasatinib in achieving MR4. 5 for newly diagnosed chronic myeloid leukemia: results of the prospective randomized phase 3 study, JALSG CML212. Blood..

[23] Nakamae H, Fujisawa S, Ogura M, Uchida T, Onishi Y, Taniwaki M (2017). Dasatinib versus imatinib in Japanese patients with newly diagnosed chronic phase chronic myeloid leukemia: a subanalysis of the DASISION 5-year final report. Int J Hematol.

[24] Nakamae H, Fukuda T, Nakaseko C, Kanda Y, Ohmine K, Ono T (2018). Nilotinib vs. imatinib in Japanese patients with newly diagnosed chronic myeloid leukemia in chronic phase: long-term follow-up of the Japanese subgroup of the randomized ENESTnd trial. Int J Hematol.

[25] Cortes JE, Apperley JF, DeAngelo DJ, Deininger MW, Kota VK, Rousselot P (2018). Management of adverse events associated with bosutinib treatment of chronic-phase chronic myeloid leukemia: expert panel review. J Hematol Oncol.

[26] Cortes JE, Saglio G, Kantarjian HM, Baccarani M, Mayer J, Boqué C (2016). Final 5-year study results of DASISION: the dasatinib versus imatinib study in treatment-naïve chronic myeloid leukemia patients trial. J Clin Oncol.

[27] Barber MC, Mauro MJ, Moslehi J (2017). Cardiovascular care of patients with chronic myeloid leukemia (CML) on tyrosine kinase inhibitor (TKI) therapy. Hematol Am Soc Hematol Educ Program.

[28] Seguro FS, Silva C, Moura CMB, Conchon M, Fogliatto L, Funke VAM (2021). Recommendations for the management of cardiovascular risk in patients with chronic myeloid leukemia on tyrosine kinase inhibitors: risk assessment, stratification, treatment and monitoring. Hematol Transfus Cell Ther.

