# Supplementary material for: Bosutinib in Japanese patients with newly diagnosed chronic-phase chronic myeloid leukemia: final 3-year follow-up results of a phase 2 study
Source: Int J Hematol. 2022 Aug 13;116(6):871–82. doi: 10.1007/s12185-022-03435-4 (PMC9668794; doi:10.1007/s12185-022-03435-4)

SUPPLEMENTARY MATERIAL

Bosutinib in Japanese patients with newly diagnosed chronic phase chronic myeloid leukemia: final 3-year follow-up results of a phase 2 study

Takaaki Ono[1] · Masayuki Hino[2] · Itaru Matsumura[3] · Shin Fujisawa[4] · Kenichi Ishizawa[5] · Emiko Sakaida[6] · Naohiro Sekiguchi[7] · Chiho Ono[8] · Mana Aizawa[9] · Yusuke Tanetsugu[9] · Yuichiro Koide[9] · Naoto Takahashi[10]

[1] Hamamatsu University Hospital, Shizuoka, Japan

[2] Osaka City University Hospital, Osaka, Japan

[3] Kindai University Hospital, Osaka, Japan

[4] Yokohama City University Medical Center, Yokohama, Japan

[5] Yamagata University Hospital, Yamagata, Japan

[6] Chiba University Hospital, Chiba, Japan

[7] National Hospital Organization Disaster Medical Center, Tokyo, Japan

[8] Pfizer Japan Inc, Tokyo, Japan

[9] Pfizer R&D Japan G.K., Tokyo, Japan

[10] Akita University Hospital, Akita, Japan

Table S1 Adverse events of special interest categories

Category	Adverse event criteria
Cardiac	• HLGTs in cardiac arrhythmias, heart failure, pericardial disorders<br>• PTs in cardiac death, sudden cardiac death, sudden death, ejection fraction decreased<br>• SMQ (narrow): Torsade de pointes/QT prolongation
Edema	• PTs contain edema, weight increased
Effusion	• PTs in pleural effusion, pericardial effusion
Gastrointestinal	• PTs in nausea, regurgitation, retching, vomiting, vomiting projectile, diarrhea, defecation urgency, frequent bowel movements, gastrointestinal hypermotility
Hemorrhage	• PTs in gastric occult blood positive, occult blood positive<br>• SMQ (narrow): hemorrhage terms (excluding laboratory terms)
Hypertension	• HLGT in vascular hypertensive disorders<br>• PTs in BP abnormal, BP ambulatory abnormal, BP ambulatory increased, BP diastolic abnormal, BP diastolic increased, BP increased, BP systolic abnormal, BP systolic increased
Infection	• SOC in infections and infestations
Liver function	• Sub SMQs (narrow): cholestasis and jaundice of hepatic origin; hepatic failure, fibrosis and cirrhosis and other liver damage–related conditions; hepatitis, non-infectious; liver-related investigations, signs and symptoms (selected relevant)<br>• PTs in ALT abnormal, ALT increased, AST abnormal, AST increased, bilirubin conjugated abnormal, bilirubin conjugated

	increased, blood bilirubin abnormal, blood bilirubin increased, blood bilirubin unconjugated increased, hepatic enzyme abnormal, hepatic enzyme increased, hepatic function abnormal, hyperbilirubinemia, hypertransaminasemia, liver function test abnormal, transaminases abnormal, transaminases increased, blood ALP abnormal, blood ALP increased, liver function test increased
Myelosuppression	• SMQs (narrow): hematopoietic cytopenias affecting >1 type of blood cell,[a] hematopoietic erythropenia, hematopoietic leukopenia, hematopoietic thrombocytopenia<br>• PTs in bone marrow toxicity, hematocrit decreased, hemoglobin decreased, hematotoxicity, anemia
Rash	• HLTs in rashes, eruptions, and exanthem NEC; erythema; acne; dermatitis and eczema
Renal	• HLT in renal failure and impairment<br>• PTs in blood creatinine abnormal, blood creatinine increased, creatinine renal clearance abnormal, creatinine renal clearance decreased, glomerular filtration rate abnormal, glomerular filtration rate decreased
Vascular	• HLGTs in coronary artery disorders; arteriosclerosis, stenosis, vascular insufficiency, and necrosis; embolism and thrombosis<br>• HLTs in arterial therapeutic procedures (excluding aortic), CNS hemorrhages and cerebrovascular accidents, CNS vascular disorders NEC, non–site specific vascular disorders NEC, peripheral vascular disorders NEC (excluding the 2 PTs flushing

	and hot flush), transient cerebrovascular events, vascular imaging procedures NEC, vascular therapeutic procedures NEC

[a]The following clustered terms for cytopenias including lymphopenia (PT = lymphopenia; lymphocyte count decreased), thrombocytopenia (PT = thrombocytopenia; platelet count decreased), anemia (PT = anemia; hemoglobin decreased), neutropenia (PT = neutropenia; neutrophil count decreased), leukopenia (PT = leukopenia; white blood cell count decreased) are used

ALP alkaline phosphatase; *ALT* alanine aminotransferase; *AST* aspartate aminotransferase; *BP* blood pressure; *CNS* central nervous system; *HLGT* High Level Group Term; *HLT* High Level Term; *NEC* not elsewhere classified; *PT* Preferred Term; *SMQ* standardized Medical Dictionary for Regulatory Activities query; *SOC* system organ class

Table S2 Major molecular response before and after dose escalation/reduction

	Bosutinib (N = 60)		
n (%)	No escalation to ≥500 mg	Dose escalation to 500 mg	Dose escalation to 600 mg
Patients with/without escalation	50 (83.3)	10 (16.7)	1 (1.7)
Attained/maintained MMR	33 (66.0)	9 (90.0)	1 (100)
First MMR following escalation	NA	2 (22.2)	1 (100)
MMR before and after escalation	NA	7 (77.8)	0
MMR before but not after escalation	NA	0	0
	No reduction to ≤300 mg	Dose reduction to 300 mg	Dose reduction to 200 mg
Patients with/without reduction	24 (40.0)	36 (60.0)	9 (15.0)
Attained/maintained MMR	19 (79.2)	23 (63.9)	3 (33.3)
First MMR following reduction	NA	17 (73.9)	2 (66.7)
MMR before and after reduction	NA	6 (26.1)	1 (33.3)
MMR before but not after reduction	NA	0	0

Modified as-treated population. Only first dose escalation/reduction to the dose level for the patient is considered in this table. MMR = ≤0.1% *BCR*::*ABL1* on the IS with ≥3000 *ABL1* transcripts assessed

IS international scale; *MMR* major molecular response; *NA* not applicable

Table S3 Cumulative molecular response rates any time on treatment by Sokal score, age, and modified Charlson comorbidity index

	Bosutinib (N = 60)							
n (%)	Low-risk Sokal score (n = 28)	Intermediate-risk Sokal score (n = 25)	High-risk Sokal score (n = 7)	<65 years (n = 41)	≥65 years (n = 19)	mCCI ≤2 (n = 42)	mCCI >2 (n = 18)	Total (N = 60)
MMR	16 (57.1)	22 (88.0)	4 (57.1)	30 (73.2)	12 (63.2)	29 (69.0)	13 (72.2)	42 (70.0)
90% CI	41.8–72.5	77.3–98.7	26.4–87.9	61.8–84.6	45.0–81.4	57.3–80.8	54.9–89.6	60.3–79.7
MR^4	13 (46.4)	16 (64.0)	3 (42.9)	21 (51.2)	11 (57.9)	19 (45.2)	13 (72.2)	32 (53.3)
90% CI	30.9–61.9	48.2–79.8	12.1–73.6	38.4–64.1	39.3–76.5	32.6–57.9	54.9–89.6	42.7–63.9
$MR^{4.5}$	11 (39.3)	15 (60.0)	3 (42.9)	19 (46.3)	10 (52.6)	18 (42.9)	11 (61.1)	29 (48.3)
90% CI	24.1–54.5	43.9–76.1	12.1–73.6	33.5–59.2	33.8–71.5	30.3–55.4	42.2–80.0	37.7–58.9

Modified as-treated population. Sokal score = low risk (<0.8); intermediate risk (0.8–1.2); and high risk (>1.2). MMR = ≤0.1% *BCR*::*ABL1* on the IS with ≥3000 *ABL1* transcripts assessed; MR^4 = ≤0.01% *BCR*::*ABL1* on the IS with ≥9800 *ABL1* transcripts assessed; $MR^{4.5}$ = ≤0.0032% *BCR*::*ABL1* on the IS with ≥30,990 *ABL1* transcripts assessed. 90% CIs based on the Brookmeyer–Crowley method

IS international scale; *mCCI* modified Charlson comorbidity index; *MMR* major molecular response; *MR* molecular response

Table S4 Summary of treatment-emergent adverse events

	Bosutinib (N = 60)	
n (%)	All-causality	Treatment-related
**All patients**		
Patients with TEAEs	60 (100)	60 (100)
Patients with serious TEAEs	15 (25.0)	13 (21.7)
Patients with maximum grade ≥3 TEAEs	49 (81.7)	47 (78.3)
Patients with maximum grade 5 TEAEs	0	0
Patients with TEAEs leading to discontinuation of bosutinib[a]	21 (35.0)	20 (33.3)
Patients with TEAEs leading to dose reduction of bosutinib[b]	37 (61.7)	36 (60.0)
Patients with TEAEs leading to temporary discontinuation of bosutinib[c]	44 (73.3)	41 (68.3)
**Patients <65 years of age**		
Patients with TEAEs	41 (100.0)	41 (100.0)
Patients with serious TEAEs	10 (24.4)	8 (19.5)
Patients with maximum grade ≥3 TEAEs	31 (75.6)	30 (73.2)
Patients with maximum grade 5 TEAEs	0	0

Patients with TEAEs leading to discontinuation of bosutinib[a]	10 (24.4)	10 (24.4)
Patients with TEAEs leading to dose reduction of bosutinib[b]	22 (53.7)	21 (51.2)
Patients with TEAEs leading to temporary discontinuation of bosutinib[c]	26 (63.4)	24 (58.5)
**Patients ≥65 years of age**		
Patients with TEAEs	19 (100.0)	19 (100.0)
Patients with serious TEAEs	5 (26.3)	5 (26.3)
Patients with maximum grade ≥3 TEAEs	18 (94.7)	17 (89.5)
Patients with maximum grade 5 TEAEs	0	0
Patients with TEAEs leading to discontinuation of bosutinib[a]	11 (57.9)	10 (52.6)
Patients with TEAEs leading to dose reduction of bosutinib[b]	15 (78.9)	15 (78.9)
Patients with TEAEs leading to temporary discontinuation of bosutinib[c]	18 (94.7)	17 (89.5)
**Patients with an mCCI score ≤2**		
Patients with TEAEs	42 (100.0)	42 (100.0)
Patients with serious TEAEs	12 (28.6)	11 (26.2)
Patients with maximum grade ≥3 TEAEs	34 (81.0)	34 (81.0)
Patients with maximum grade 5 TEAEs	0	0

Patients with TEAEs leading to discontinuation of bosutinib[a]	14 (33.3)	14 (33.3)
Patients with TEAEs leading to dose reduction of bosutinib[b]	28 (66.7)	27 (64.3)
Patients with TEAEs leading to temporary discontinuation of bosutinib[c]	29 (69.0)	29 (69.0)
**Patients with an mCCI score >2**		
Patients with TEAEs	18 (100.0)	18 (100.0)
Patients with serious TEAEs	3 (16.7)	2 (11.1)
Patients with maximum grade ≥3 TEAEs	15 (83.3)	13 (72.2)
Patients with maximum grade 5 TEAEs	0	0
Patients with TEAEs leading to discontinuation of bosutinib[a]	7 (38.9)	6 (33.3)
Patients with TEAEs leading to dose reduction of bosutinib[b]	9 (50.0)	9 (50.0)
Patients with TEAEs leading to temporary discontinuation of bosutinib[c]	15 (83.3)	12 (66.7)

As-treated population. Serious TEAEs were according to the investigator's assessment. [a]Patients whose record indicated that the TEAE caused them to be discontinued from bosutinib treatment; [b]Patients whose record indicated that action taken with bosutinib was dose reduction; [c]Patients whose record indicated that action taken with bosutinib was temporary discontinuation

TEAE treatment-emergent adverse event

Table S5 Treatment-emergent adverse events leading to treatment discontinuations by year, all causalities

MedDRA PT, *n* (%)	Bosutinib (*N* = 60)				
	Year 1 (*N* = 60)	Year 2 (*N* = 42)	Year 3 (*N* = 39)	≥Year 4 (*N* = 31)	Total (*N* = 60)
Any TEAE	17 (28.3)	3 (7.1)	1 (2.6)	0	21 (35.0)
ALT increased	6 (10.0)	0	0	0	6 (10.0)
AST increased	5 (8.3)	0	0	0	5 (8.3)
Drug eruption	2 (3.3)	0	0	0	2 (3.3)
Erythema multiforme	2 (3.3)	0	0	0	2 (3.3)
Lipase increased	2 (3.3)	0	0	0	2 (3.3)
Neutropenia	1 (1.7)	0	0	0	1 (1.7)
Thrombocytopenia	1 (1.7)	0	0	0	1 (1.7)
Drug-induced liver injury	1 (1.7)	0	0	0	1 (1.7)
Liver disorder	1 (1.7)	0	0	0	1 (1.7)
Pneumonia	1 (1.7)	0	0	0	1 (1.7)
Blood creatinine increased	0	0	1 (2.6)	0	1 (1.7)

Hepatic enzyme increased	1 (1.7)	0	0	0	1 (1.7)
Pancreatic enzymes increased	1 (1.7)	0	0	0	1 (1.7)
Breast cancer	0	1 (2.4)	0	0	1 (1.7)
Nephropathy toxic	0	1 (2.4)	0	0	1 (1.7)
Pleural effusion	0	1 (2.4)	0	0	1 (1.7)

As-treated population. One year = 365.25 days. Patients were counted only once per event. MedDRA version 23.1 coding dictionary applied.

The following clustered terms for cytopenias are used: neutropenia (PT = neutropenia; neutrophil count decreased), and thrombocytopenia (PT = thrombocytopenia; platelet count decreased)

ALT alanine aminotransferase; *AST* aspartate aminotransferase; *MedDRA* Medical Dictionary for Regulatory Activities; *PT* Preferred Term; *TEAE* treatment-emergent adverse event

Table S6 Follow-up CML treatment

n (%)	Bosutinib (*N* = 60)
Any follow-up CML treatment	24 (40.0)
Bosutinib	4 (6.7)
Dasatinib	11 (18.3)
Imatinib	7 (11.7)
Nilotinib	9 (15.0)
Ponatinib	2 (3.3)

Three patients out of 24 above discontinued bosutinib due to reasons other than adverse events: one patient due to change hospital (subsequent bosutinib), one patient due to breast cancer (subsequent bosutinib), and one patient due to physician decision (subsequent dasatinib, nilotinib and ponatinib)

CML chronic myeloid leukemia

Table S7 Characteristics of treatment-emergent adverse events of special interest

	Bosutinib (N = 60)										
n (%)	Liver function TEAEs[a]	Increased ALT[b]	Increased AST[b]	Gastro-intestinal TEAEs[a]	Diarrhea[b]	Myelo-suppression TEAEs[a]	Rash TEAEs[a]	Cardiac TEAEs[a]	Vascular TEAEs[a]	Hypertension TEAEs[a]	Renal TEAEs[a]
Total patients with event	48 (80.0)	33 (55.0)	28 (46.7)	52 (86.7)	52 (86.7)	27 (45.0)	34 (56.7)	5 (8.3)	1 (1.7)	4 (6.7)	10 (16.7)
Medical history of event	4 (8.3)	2 (6.1)	1 (3.6)	0	0	7 (25.9)	5 (14.7)	0	0	2 (50.0)	1 (10.0)
Time to first event, median (range),[c] days	15.0 (1–169)	15.0 (1–169)	15.0 (1–57)	1.0 (1–271)	1.0 (1–271)	21.0 (1–99)	24.5 (6–1093)	505.0 (24–799)	41.0 (41–41)	598.0 (379–736)	671.0 (7–925)
Duration of any grade event,[d] median (range), days	14.0 (1–1002)	14.0 (2–1002)	14.0 (2–211)	3.0 (1–824)	3.0 (1–824)	14.0 (2–497)	27.0 (2–815)	42.0 (8–337)	0	91.0 (91–91)	56.0 (3–421)
Duration of treatment stop events,[e] median (range), days	18.5 (8–48)	14.0 (8–28)	20.0 (13–28)	9.0 (1–28)	8.0 (1–28)	14.0 (6–48)	6.0 (6–8)	0	0	0	53.0 (53–53)
Treatment change[f]											
Dose reduction	17 (35.4)	13 (39.4)	8 (28.6)	6 (11.5)	3 (5.8)	7 (25.9)	4 (11.8)	4 (80.0)	0	0	1 (10.0)

Temporary interruption	29 (60.4)	17 (51.5)	10 (35.7)	8 (15.4)	7 (13.5)	10 (37.0)	4 (11.8)	0	0	0	1 (10.0)
Treatment rechallenge[g]	28 (96.6)	17 (100)	9 (90.0)	8 (100)	7 (100)	10 (100)	4 (100)	0	0	0	1 (100.0)
Successful treatment rechallenge[h]	20 (71.4)	13 (76.5)	6 (66.7)	8 (100)	7 (100)	10 (100)	4 (100)	0	0	0	0
Permanent treatment discontinuation	10 (16.7)	6 (10.0)	5 (8.3)	0	0	2 (3.3)	0	0	0	0	1 (1.7)
Treated with concomitant medication	27 (56.3)	16 (48.5)	14 (50.0)	50 (96.2)	50 (96.2)	2 (7.4)	28 (82.4)	2 (40.0)	1 (100.0)	4 (100.0)	3 (30.0)

As-treated population. *n* (%) unless otherwise stated. [a]TEAE cluster; [b]MedDRA PT; [c]Time to event for a given patient was calculated as (start date of first TEAE – date of first bosutinib dose) + 1 for non-missing or non-partial dates; [d]Duration was defined as (stop date of TEAE – start date of TEAE) + 1 for non-missing or non-partial dates; [e]Duration is based on distinct treatment stop events from bosutinib data that were triggered by the TEAE under consideration; [f]Patients with multiple TEAEs reported were counted in each category; [g]Rechallenge is defined as patients who had any grade toxicity TEAEs leading to temporary stop of bosutinib and who were subsequently redosed; [h]Successful is defined as patients redosed after the specific TEAE type who have no subsequent TEAE of that type or are not discontinued permanently due to that type of TEAE

ALT alanine aminotransferase; *AST* aspartate aminotransferase; *MedDRA* Medical Dictionary for Regulatory Activities; *PT* Preferred Term; *TEAE* treatment-emergent adverse event

Table S8 Cardiac, vascular, hypertension, and renal adverse events of special interest

	Bosutinib (N = 60)			
	All-causality		Treatment-related	
n (%)	Any grade	Grade ≥3	Any grade	Grade ≥3
Cardiac TEAEs	5 (8.3)	0	5 (8.3)	0
Pericardial effusion	3 (5.0)	0	3 (5.0)	0
Cardiac failure	2 (3.3)	0	2 (3.3)	0
Vascular TEAEs	1 (1.7)	0	0	0
Peripheral coldness	1 (1.7)	0	0	0
Hypertension TEAEs	4 (6.7)	1 (1.7)	3 (5.0)	1 (1.7)
Hypertension	4 (6.7)	1 (1.7)	3 (5.0)	1 (1.7)
Renal TEAEs	10 (16.7)	1 (1.7)	9 (15.0)	0
Increased blood creatinine	6 (10.0)	0	5 (8.3)	0
Acute kidney injury	2 (3.3)	0	2 (3.3)	0
Chronic kidney disease	1 (1.7)	1 (1.7)	0	0
Renal impairment	1 (1.7)	0	1 (1.7)	0
Renal injury	1 (1.7)	0	1 (1.7)	0

As-treated population.

TEAE treatment-emergent adverse event

Fig. S1 Cumulative incidence of molecular response: (**a**) MMR, (**b**) MR^4, and (**c**) $MR^{4.5}$. Modified as-treated population. Molecular response was analyzed using cumulative incidence, adjusting for the competing risk without response. MMR = ≤0.1% *BCR::ABL1* on the IS with ≥3000 *ABL1* transcripts assessed. MR^4 = ≤0.01% *BCR::ABL1* on the IS with ≥9800 *ABL1* transcripts assessed. $MR^{4.5}$ = ≤0.0032% *BCR::ABL1* on the IS with ≥30,990 *ABL1* transcripts assessed. 90% CIs based on delta method with the log(-log) transformation. *CI* confidence interval; *IS* international scale; *MMR* major molecular response; *MR* molecular response

(a) MMR

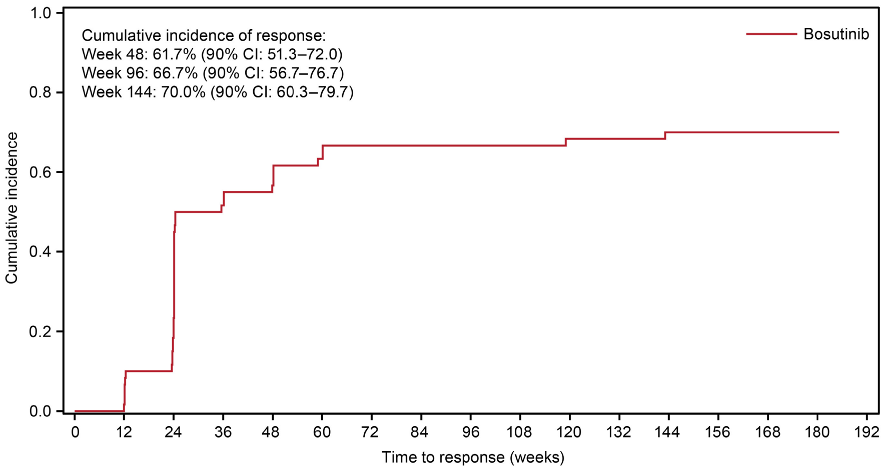

Number at risk

0	12	24	36	48	60	72	84	96	108	120	132	144	156	168	180	192
60	56	35	13	9	5	3	3	3	3	2	2	1	1	1	1	0

(b) MR^4

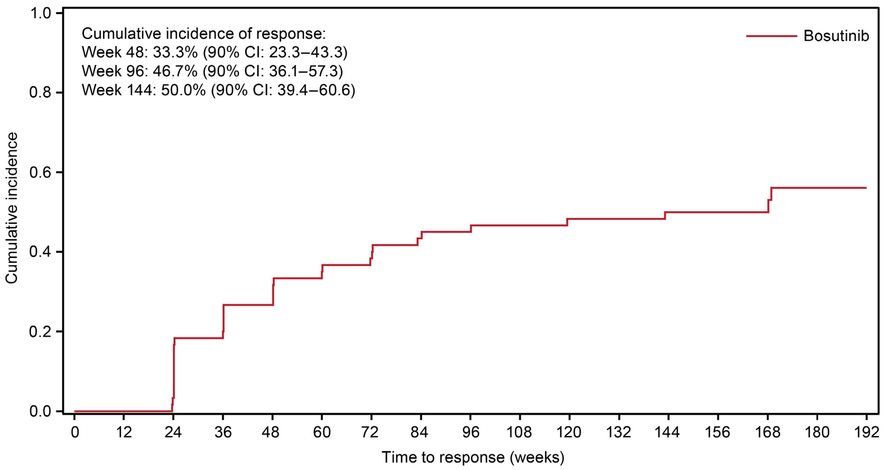

Number at risk

0	12	24	36	48	60	72	84	96	108	120	132	144	156	168	180	192
60	56	44	33	26	22	19	15	14	13	12	12	11	8	6	4	1

(c) $MR^{4.5}$

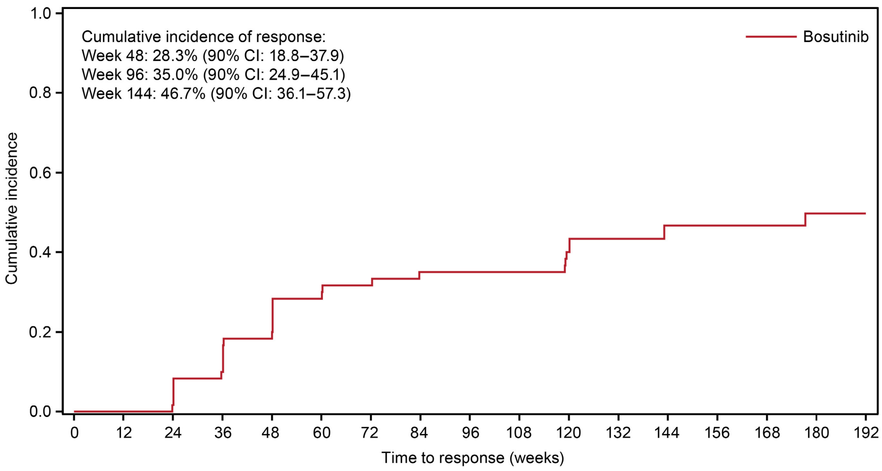

Number at risk

0	12	24	36	48	60	72	84	96	108	120	132	144	156	168	180	192
60	56	45	38	31	25	23	20	20	20	17	15	13	10	7	6	1

Fig. S2 Cumulative incidence of event-free survival on treatment. Modified as-treated population. Event-free survival was analyzed using cumulative incidence, adjusting for the competing risk events (treatment discontinuation for reasons other than progressive disease). 90% CIs based on delta method with the log(-log) transformation.
CI confidence interval; *EFS* event-free survival

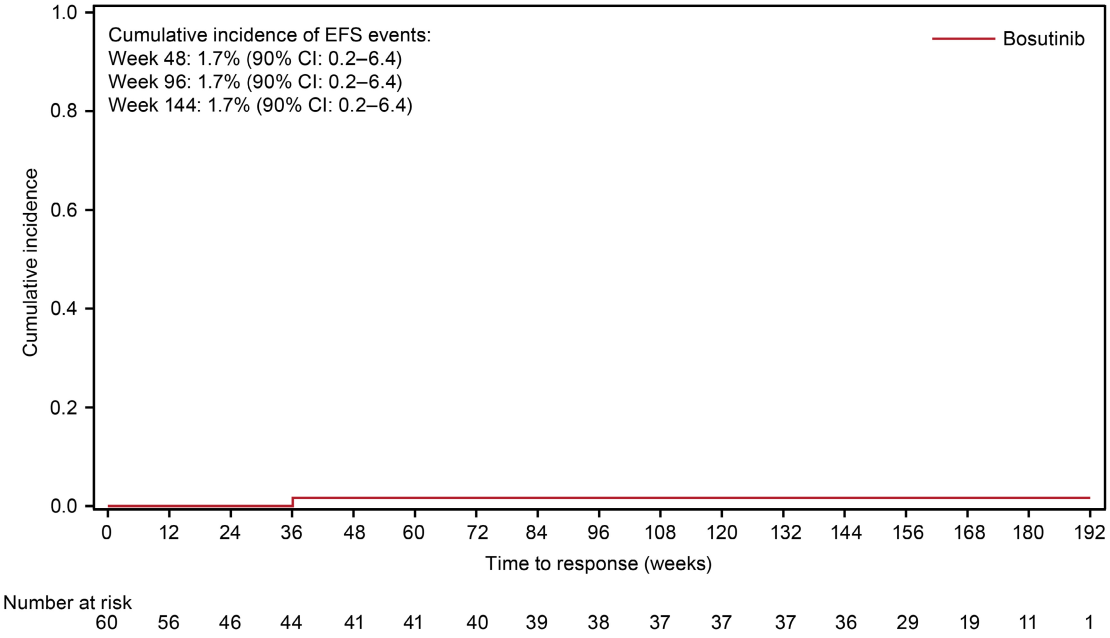

Fig. S3 Kaplan–Meier plot of overall survival. Modified as-treated population. 90% CIs based on Greenwood's log(-log) transformation. *CI* confidence interval

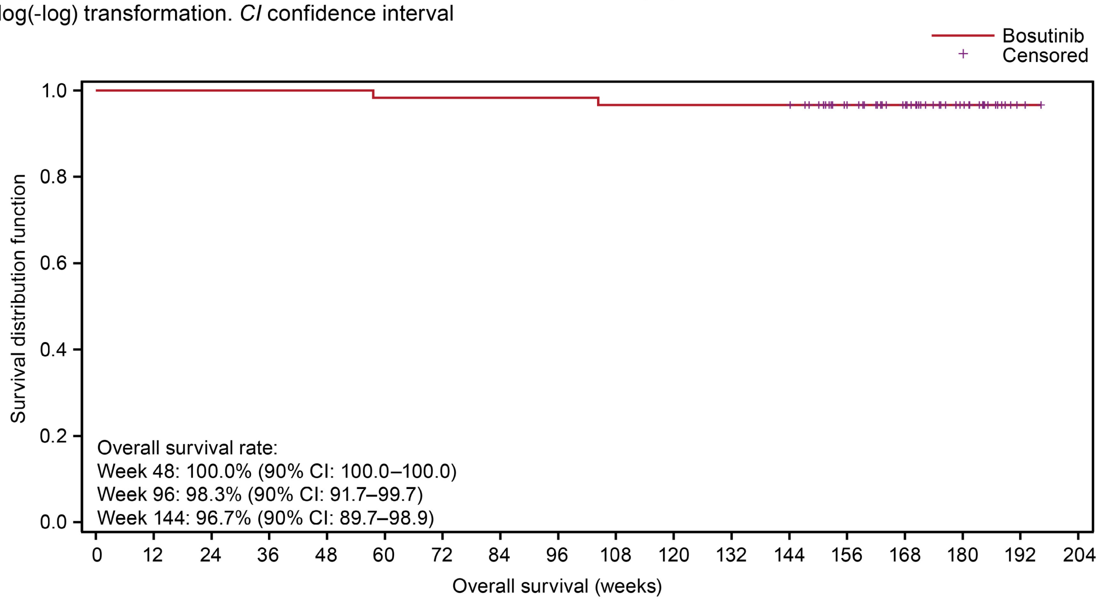

Number at risk

60	60	60	60	60	59	59	59	59	58	58	58	58	47	36	19	2	0

Fig. S4 Cumulative incidence of molecular response by Sokal score: (**a**) MMR, (**b**) MR^4, and (**c**) $MR^{4.5}$. Modified as-treated population. Molecular response was analyzed using cumulative incidence, adjusting for the competing risk without response. MMR = ≤0.1% *BCR::ABL1* on the IS with ≥3000 *ABL1* transcripts assessed. MR^4 = ≤0.01% *BCR::ABL1* on the IS with ≥9800 *ABL1* transcripts assessed. $MR^{4.5}$ = ≤0.0032% *BCR::ABL1* on the IS with ≥30,990 *ABL1* transcripts assessed. *IS* international scale; *MMR* major molecular response; *MR* molecular response

(a) MMR

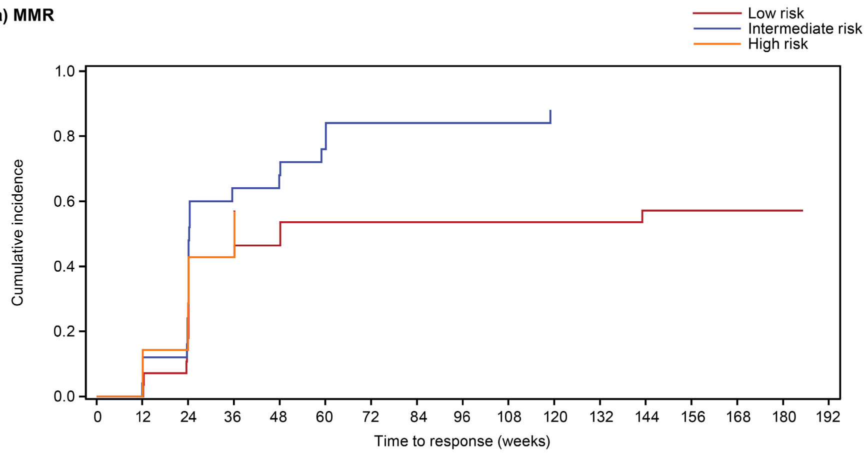

Number at risk																	
Low risk	28	25	16	6	4	2	2	2	2	2	2	2	1	1	1	1	0
Intermediate risk	25	25	16	6	5	3	1	1	1	1	0	0	0	0	0	0	0
High risk	7	6	3	1	0	0	0	0	0	0	0	0	0	0	0	0	0

(b) MR^4

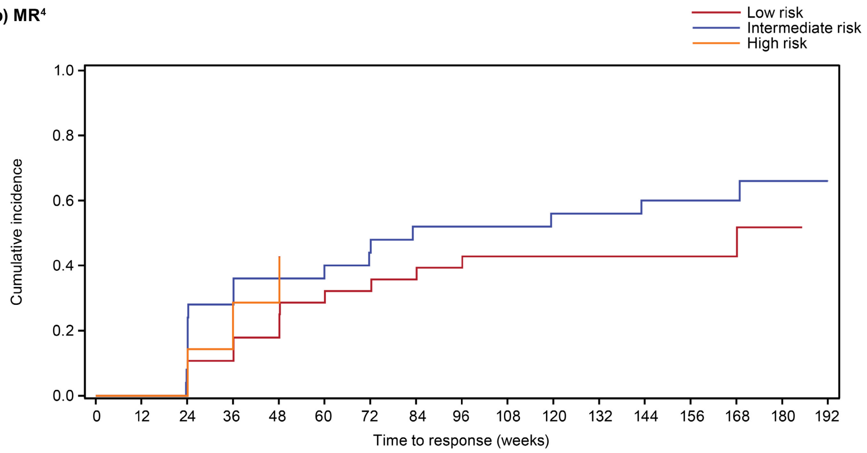

Number at risk																	
Low risk	28	25	20	15	12	9	8	7	6	5	5	5	5	3	2	1	0
Intermediate risk	25	25	20	15	13	13	11	8	8	8	7	7	6	5	4	3	1
High risk	7	6	4	3	1	0	0	0	0	0	0	0	0	0	0	0	0

(c) $MR^{4.5}$

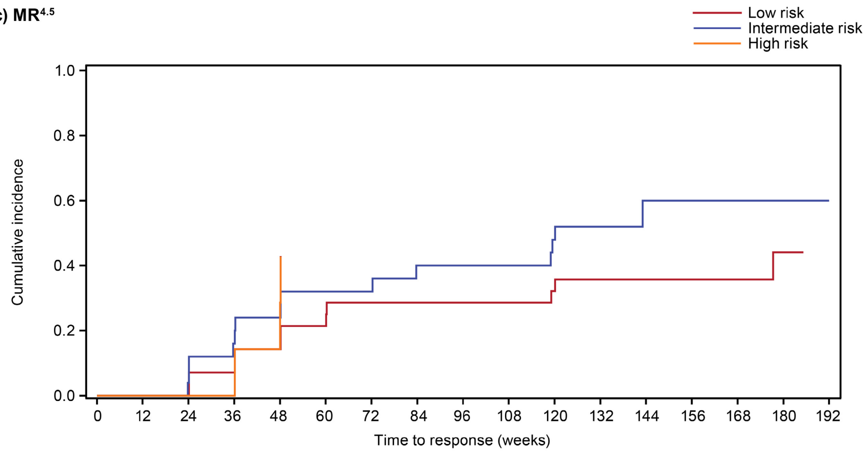

Number at risk																	
Low risk	28	25	20	16	13	11	9	9	9	9	8	7	7	5	3	2	0
Intermediate risk	25	25	21	18	16	14	14	11	11	11	9	8	6	5	4	4	1
High risk	7	6	4	4	2	0	0	0	0	0	0	0	0	0	0	0	0

Fig. S5 All grade and grade ≥3 treatment-emergent adverse events (≥20%) by subgroup: (**a**) age and (**b**) modified Charlson comorbidity index. As-treated population. *ALT* alanine aminotransferase; *AST* aspartate aminotransferase; *GGT* gamma-glutamyltransferase; *mCCI* modified Charlson comorbidity index; *TEAE* treatment-emergent adverse event

(a) Age

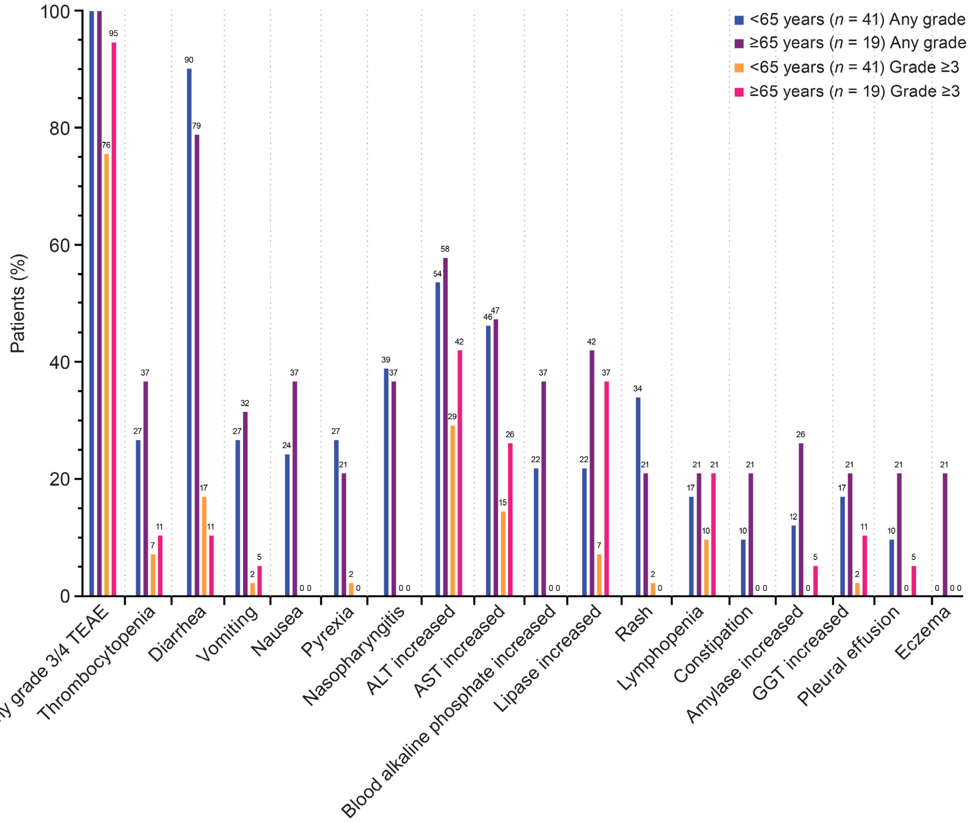

(b) mCCI

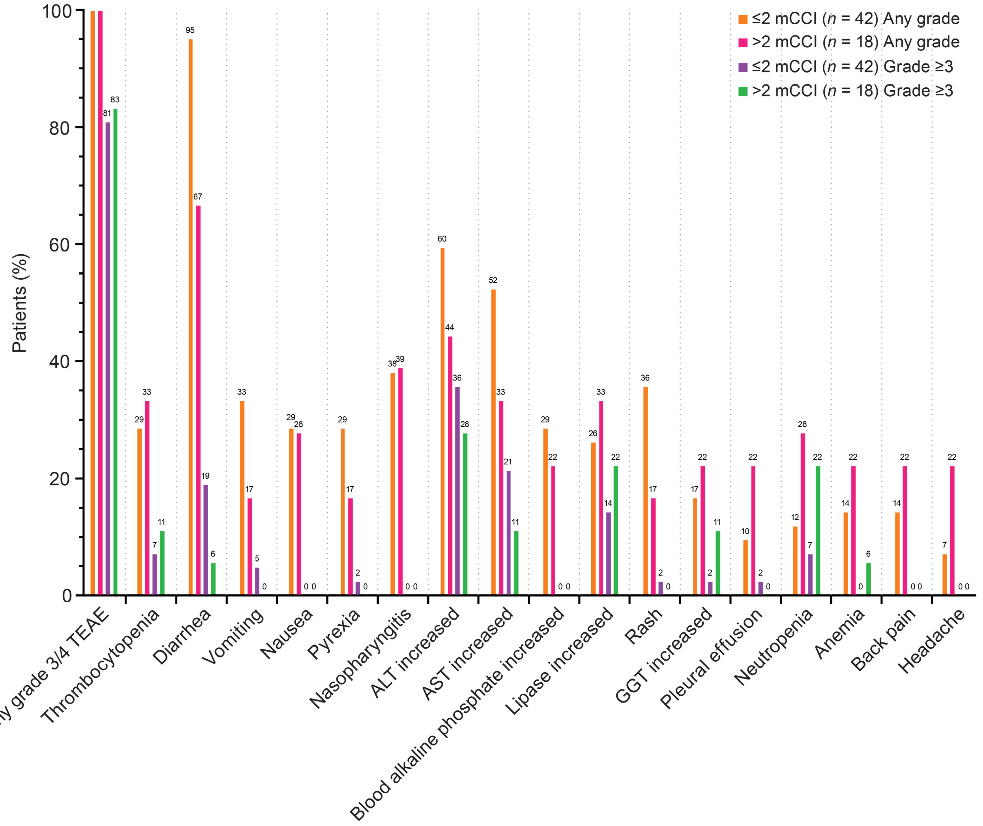

Supplement: Supplementary file 1 — Supplementary file1 (PDF 4896 KB) [file 12185_2022_3435_MOESM1_ESM.pdf]
